# Reviewed article: Carvalho EA, Hopker RH, Pedroso GH, Almeida LS, Pacher JAT, Antônio ALM, Souza J, Zeny MS, Santos MLSF, Valle DAV, Andrade FA. Characterization of patients treated at a rare disease referral service: a descriptive study, 2016-2021. Epidemiol Serv Saúde. 2024;33:e20240204. 10.1590/S2237-96222024v33e20240204.en

**DOI:** 10.1590/S2237-96222024v33e20240204.a

**Published:** 2024-12-20

**Authors:** Cassio Hartmann

**Affiliations:** 1 Instituto Federal de Alagoas, Macéio, AL, Brazil

## Peer review A

## Questionnaire

Does the manuscript contain new and significant information to justify publication? Yes

Does the Abstract (Summary) clearly and accurately describe the content of the article? Yes

Is the problem significant and concisely stated? Yes

Are the methods described comprehensively? Yes

Are the interpretations and conclusions justified by the results? Yes

Is adequate reference made to other work in the field? Yes

## Manuscript Structure

Length of article is: Adequate

Number of tables is: Adequate

Number of figures is: Adequate

**Please state any conflict(s) of interest that you have in relation to the review of this paper (state “none” if this is not applicable).**

None

**Interest:** Excellent

**Quality:** Good

**Originality:** Good

**Overall:** Good

**Recommendation:** Accept

## Comments

Parabéns aos autores (as) pela escolha do tema, por ser uma área que carece de informações e de profissíonais das áreas de saúde, para trabalhar com os raros!

